# The Fibromyalgia Pain Experience: A Scoping Review of the Preclinical Evidence for Replication and Treatment of the Affective and Cognitive Pain Dimensions

**DOI:** 10.3390/biomedicines12040778

**Published:** 2024-04-02

**Authors:** Cassie M. Argenbright, Alysia M. Bertlesman, Izabella M. Russell, Tracy L. Greer, Yuan B. Peng, Perry N. Fuchs

**Affiliations:** 1 Department of Psychology and Biobehavioral Sciences, St. Jude Children’s Research Hospital, Memphis, TN 38105, USA; 2 Department of Psychology, The University of Texas at Arlington, Arlington, TX 76019, USA; alysia.bertlesman@mavs.uta.edu (A.M.B.); imr6096@mavs.uta.edu (I.M.R.); tracy.greer@uta.edu (T.L.G.); ypeng@uta.edu (Y.B.P.); 3 Department of Psychological Science, The University of Texas Rio Grande Valley, Edinburg, TX 78539, USA; perry.fuchs@utrgv.edu

**Keywords:** fibromyalgia, preclinical, affect, cognition, sleep, chronic pain

## Abstract

Fibromyalgia is a chronic, widespread pain disorder that is strongly represented across the affective and cognitive dimensions of pain, given that the underlying pathophysiology of the disorder is yet to be identified. These affective and cognitive deficits are crucial to understanding and treating the fibromyalgia pain experience as a whole but replicating this multidimensionality on a preclinical level is challenging. To understand the underlying mechanisms, animal models are used. In this scoping review, we evaluate the current primary animal models of fibromyalgia regarding their translational relevance within the affective and cognitive pain realms, as well as summarize treatments that have been identified preclinically for attenuating these deficits.

## 1. Pain as a Sensory, Affective and Cognitive Experience

In 2020, the International Association for the Study of Pain introduced a revised definition of pain: “An unpleasant sensory and emotional experience associated with, or resembling that associated with, actual or potential tissue damage” [1]. This definition validates the experience of pain of unknown origin that may be characterized by a lack of actual or potential tissue damage, as is the case in many idiopathic pain disorders. Idiopathic pain is commonly understood to be a type of pain that has no specific or determinable cause, or which has multiple etiologies, meaning the contributions to the experience of pain may be recognized from a combination of biopsychosocial components [2].

**Figure 1:**
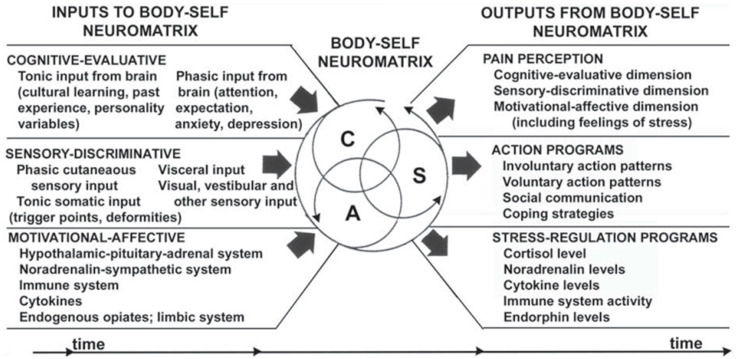
“Factors that contribute to the patterns of activity generated by the body-self neuromatrix, which comprises sensory, affective, and cognitive neuromodules. The output patterns from the neuromatrix produce the multiple dimensions of pain experience as well as concurrent homeostatic and behavioral responses”. © American Dental Education Association. Reproduced with permission from [5].

This definition of pain is crucial in that it approaches pain from a sensory and affective standpoint. Melzack and Casey [3] discussed the multidimensionality of the pain experience by addressing it across three facets: sensory-discriminative, affective-motivational, and cognitive-evaluative. “Pain” is a label for many different subjective experiences, wherein there are variations in magnitude and intensity across the sensory-discriminative and motivational-affective dimensions, intricately intertwined with cognitive modulation and appraisal of the experience. Due to this very complex relationship, it can be understood that pain does not exist in any one singular realm. The individual neuromatrix, comprised of each of these dimensions, would be responsible for giving rise to the behavioral characteristics used to categorize what we understand to be “pain behavior”, such as aversive drive, sensory modulation, and appraisal of a pain state [3,4,5]. Understanding these contributions from each realm and how they give rise to behavior is important in translationally evaluating pain states, because attempting to treat pain from a unidimensional approach, such as focusing solely on the sensory-discriminative realm, does not offer improvement with respect to the entire pain experience [3]. This creates additional challenges for etiological understanding of the pain state and further supports the need to integrate information from the affective and cognitive experiences of patients with idiopathic pain disorders to develop actionable phenotypes that may lead to more precise and thorough treatments (Figure 1).

A translational approach is key to gaining a better understanding of the differing dimensions contributing to the pain experience and ultimately the approaches that may best treat pain. Preclinical trials are a critical contributor to the development of potentially successful treatments into marketed medications and management approaches [6]. There are various methodologies for evaluating the affective, cognitive, and sensory dimensions of pain in animals. With respect to measuring affect, Mendl and Paul [7] provide an operational definition for affective experimental investigations into non-human species:

“Animal affective states are elicited by rewards and punishers or their predictors. A reward is anything for which an animal will work, and a punisher is anything that it will work to escape or avoid. Rewards or the absence of punishers, and associated predictions thereof, induce positive affect. Punishers or the absence of rewards, and associated predictions thereof, induce negative affect. Short-term emotion-like states follow immediately from individual rewarding or punishing events, whilst cumulative experience of events influences longer-term mood-like states” [7].

Applying this operationalization to pain affect formulates the experience of pain as a homeostatic emotion that may be approached in the same manner as drive-reduction theory. When pain is experienced (i.e., drive) it disrupts homeostasis (i.e., need) and creates an unpleasant state wherein an animal is motivated to respond behaviorally [8]. Measuring this behavioral response to punishers and rewards is the overarching methodological goal employed to quantify pain affect.

The homeostatic disruption of a pain state demands attention to achieve resolution [8]. To resolve, maintain, or revert to homeostatic function, an organism must make decisions because of the pain state. Additionally, the cognitive modulation of affective and sensory pain implies that decisions and responses to rewards or punishers in the environment are a result of appraisal of said multidimensional pain state, alongside activation of memory cues associated with the promotion of survival [3]. More simply, “good” decision-making in animals must result from cognitively appraising costs, benefits, and consequences associated with rewards and punishers in a manner that promotes normal homeostatic function [8]. This multifaceted contribution to a behavioral response during the experience of pain is well-established, as there is evidence for pain states producing cognitive impairment in animals [9,10]

The sensory dimension of pain behaviors is displayed in a myriad of ways, dependent upon the type of pain experienced. The sensory processing of a physical pain state is a necessity, given that the processing of pain on a neural level serves as the foundation for the confirmation that pain is being experienced. This somatic input, and the motivational and evaluative appraisals of it, mapped throughout the brain gives rise to the concept of the individual pain neuromatrix [4], which is modified through experience and provides sensational perceptions.

The interrelation between the affective, cognitive, and sensory dimensions of pain is complex. The state of homeostatic disruption induced by pain produces motivationally based behaviors and variations in cognitive states or evaluations because of somatic nociceptive processing within the neuromatrix [4,5]. While the sensation component is a necessity for the experience of pain, approaching the study of pain solely within the sensory dimension is insufficient, given the experience spans into other dimensions of the body-self neuromatrix [3,4,5]. Approaching pain as a multidimensional phenomenon proves advantageous, particularly in painful manifestations where underlying somatic mechanisms are elusive. In these cases, the affective and cognitive dimensions serve to inform the experience of the pain state, and often contribute to diagnosis and management of many idiopathic pain disorders, such as fibromyalgia. 

## 2. Fibromyalgia

Upwards of 2% of the U.S. population suffers from fibromyalgia (FMS) and women are twice as likely to be diagnosed [11]. FMS has no single identifiable etiology nor robustly efficacious treatment, which has contributed to skyrocketing costs and productivity loss on both a national and individual level [12,13]. There is a growing body of research focused on identifying the potential underlying biological components contributing to the development of the FMS experience (see [14] for review). While many studies have identified valuable underlying biological markers that can be used to recognize factors that may be contributing to the FMS experience, a lack of consistent identifiable contributors to FMS among patients has provided a challenge in validating etiological theories of FMS. Importantly, this gap in our understanding of the etiology of FMS underscores the need to synthesize multiple approaches to identify how FMS develops and is expressed across individuals.

### 2.1. Fibromyalgia Diagnosis and Characterization

Understanding the affective and cognitive experiences of FMS pain is crucial and has gone so far as to impact the ACR diagnostic criteria, where FMS diagnosis in adults now includes a widespread pain index (WPI) score ≥ 7 and a symptom severity (SS) score ≥ 5, or a WPI score of 4–6 and a SS score ≥ 9 [15]. The inclusion of the SS score in the primary FMS criteria serves to emphasize the affective nature of FMS, such that patients must display a minimum level of negative emotionality, cognitive deficits, or sleep disturbances to meet criteria for ACR diagnosis. SS scale scores are valuable and have been correlated with higher anxiety and depression diagnoses, as well as higher pain scores and rates of sleep disturbances [16,17]. Lifelong rates of anxiety and depression are significantly higher within the FMS population compared to controls, with FMS populations in some countries being at nearly ten-fold higher risk for suicide [18]. Cognitive dysfunction, coined “fibro fog”, is another common feature of this disorder, with patients reporting subjective deficits in executive function, working memory, semantic memory, episodic memory, and attention [17,18,19], that have been objectively confirmed with experimental tasks [20]. There is substantial evidence supporting the idea that healthy sleep modulates and integrates these affective and evaluative processes [21], but additional research has documented deficits in sleep quality, duration, latency, and efficiency among FMS patients [22]. This intricate relationship between alterations in cognitive and affective mechanisms, which may be related to disturbances in sleep patterns, substantiates the understanding of FMS as a multifaceted pain experience.

### 2.2. Fibromyalgia Treatment

The majority of current FMS treatment methodologies employ a multidisciplinary approach [18,23]. There are three FDA-approved drugs for the treatment of FMS: duloxetine (Cymbalta^®^), milnacipran (Savella^®^), and pregabalin (Lyrica^®^) [23,24]. While the American College of Rheumatology [24] strongly urges against the use of opioids, acetaminophen, and NSAIDs for FMS, other common treatment approaches employ anticonvulsants, muscle relaxants, antipsychotics, low dose analgesics, hypnotics, and cannabis [18,25]. Off-label antidepressants are also frequently employed and have shown specific benefits. For example, a meta-analysis revealed that duloxetine was the most effective of FDA-approved drugs for improving pain and depression, while amitriptyline, an off-label FMS pharmacologic, was most effective for improving sleep, fatigue, and patient quality of life [26]. Despite some benefits to pain management, a primary concern with many of these treatment methodologies is that they are dependent on the individual pain profile; many treatments only selectively alleviate symptoms and simultaneously are associated with many adverse effects [17,18]. Furthermore, many of these treatments are not financially accessible or pose a significant economic burden to patients [27].

However, the European Alliance of Associations for Rheumatology (EULAR) [25] and the ACR [24] offer additional recommendations for FMS management, with the strongest suggestions geared towards aerobic and strength exercise regimens, cognitive-behavioral therapy (CBT), acupuncture, hydrotherapy, chiropractic, meditation and mindfulness, and sleep hygiene [25]. Of the more readily available management strategies such as patient education and exercise therapies, review of the literature has identified that these approaches primarily show evidence for short-term alleviation of FMS pain, fatigue, depression, and anxiety [28]. Overall, there is a need for a long-term, well-rounded approach to FMS treatment that improves manifestations of this pain disorder across several dimensions of the disorder. Furthermore, as mentioned previously, greater understanding of the mechanisms involved in multiple dimensions of FMS are needed to better align treatments with specific symptom profiles.

### 2.3. Purpose

With this affective and cognitive characterization of FMS, there should be a shift away from preclinical replication of FMS pain on solely a sensory or biological level and movement towards including affective and cognitive replication. The cognitive and affective dimensions of FMS pain are no longer “comorbidities”, as comorbidities are understood to exist simultaneously and independently of a condition. Rather, these dimensions of pain within the FMS pain experience are simultaneous and interdependent, as emphasized by Melzack and Casey [3], and thus should be treated as such on a preclinical level. Considering how crucial animal models are to effective investigation of underlying neurobiological processes of pain and its treatment, creating an animal model that simply mimics a sensory experience reported by FMS patients and classifying it as a model of FMS pain is unrealistic. Clinical pain manifestations are not to be diagnosed as FMS by simply reporting negative sensory experiences, unaccompanied by deficits in affective and cognitive processing. Therefore, in order to effectively evaluate neurobiological theories and treatment for FMS on a preclinical level, it is crucial to understand how well animal models of FMS replicate the multidimensional manifestation of this disorder. Therefore, this paper provides a review of the primary preclinical models of FMS in their success in replicating pain on an affective and cognitive level, while further identifying effective treatment methodologies for improving negative affect and cognitive deficits on a preclinical level.

## 3. Preclinical Models of Fibromyalgia

For the scope of this review, articles assessing the efficacy of various animal models of FMS were identified using a semi-systematic process. The original publications for the following models were identified in PubMED and Google Scholar: reserpine [29], acidic saline [30], fatigue-enhanced muscle pain [31,32], cold stress [33], sound stress [34,35], and subchronic swim stress [36]. Within the “cited by” classification in each database, articles that were able to be located in English were included when the initial assessment of the methods: (1) utilized an experimental preclinical design, (2) employed the original model as cited or within a comparable methodology, and (3) reported data from a minimum of one paradigm assessing preclinical affect, cognition, or sleep. Inspection of the literature identified 2069 articles to be assessed across the inclusion process. Following a secondary inclusion analysis, a total of 90 articles were included for review. Included articles are categorized by FMS-like pain model use, and subcategorized by investigations of affect, cognition, and sleep. Table A1 (Appendix A) offers a summarization of each of the behavioral measures and treatments addressed within each included study, as well as their significance in contributing to the replication of the affective and cognitive FMS pain dimensions.

### 3.1. Reserpine

The reserpine model of FMS, or the biogenic amine depletion model, developed by Nagakura, Oe, Aoki, and Matsuoka [29], has gained traction within preclinical investigations of FMS due to its reported efficacy in inducing hyperalgesia accompanied by depression-like behaviors in animals. Initial investigations employed subcutaneous injections of reserpine (0.1 mg/kg, 0.3 mg/kg, or 1 mg/kg) daily, for three consecutive days, in male and female rats (8-week-old Sprague-Dawley, 240–300 g and 160–190 g, respectively). The original development of the model included investigations of muscle withdrawal thresholds (MWTs), mechanical paw withdrawal thresholds (MPWTs), forced swim test (FST), and biogenic amine contents within the spinal cord, thalamus, and prefrontal cortex. Nagakura et al. [29] further investigated the pharmacological profile of the model by investigating the impact of pregabalin (p.o.), duloxetine (p.o.), pramipexole (s.c.), and diclofenac (p.o.) on MWT and MPWT restoration. Across both male and female animals, 1 mg/kg administration of reserpine produced the most pronounced reduction in evoked thresholds. FST analyses, conducted only in male rats, displayed an increase in immobility time beginning day-3 post-injection and lasting until day-14 post-injection. Dopamine (DA), serotonin (5-HT), and norepinephrine (NE) depletion across the spinal cord, thalamus, and prefrontal cortex tissues was the most prominent among 1 mg/kg reserpinized rats. Pregabalin (10 or 30 mg/kg) and pramipexole (0.3 or 1 mg/kg) administration increased MWTs, and MPWTs while duloxetine (30 mg/kg) increased MPWTs. Diclofenac failed to produce any changes in MWTs or MPWTs. Overall, the original development of this model produced biogenic amine depletion, accompanied by behavioral representations of the pain-depression dyad, and appropriately mimicked the pharmacological profile associated with FMS.

The original development of this model provides an important opportunity for the use of animals towards the investigation of the pain-depression dyad via underlying CNS mechanisms. Further evaluation of the reserpine model as a potential multidimensional approach to preclinical FMS research serves to inform clinical understanding, and thus is of prime importance to translational science [37]. 

#### Reserpine Evaluation

A significant body of literature was identified investigating affect and cognition within the reserpine model of FMS-like pain (Table 1). All identified studies in this review utilizing FST produced indications of depression-like behavior in animals as a result of the model [38,39,40,41,42,43,44,45,46,47,48,49,50,51,52,53,54,55,56,57,58,59,60,61,62,63,64,65,66,67,68,69,70]. However, several studies utilized FST as a sole measure of affect [38,39,40,41,42,43,44,45,46,47,48]. While FST is understood to evaluate behavioral despair, which may be related to concepts of learned helplessness [71], these should not be considered unconditionally when employed as a lone measure of affective function. Additional data for depression-like behaviors were identified with the Tail Suspension Test (TST) paradigm [49,51,58,66], although results failing to find these effects were also highlighted [72]. Results from Sucrose Preference Test (SPT) and Novelty Suppressed Feeding Test (NSFT) assays remained consistent, such that the model reliably produced depression-like behaviors in animals as elucidated in these assessments [37,53,56,63,66,67,73]. Further investigation into depression-like behavior using the splash test yielded mixed results, with one study reporting reduced grooming behaviors [56], while others did not [53,64]. In measures of depression-like behavior, the reserpine model appears to be effective in inducing behavioral changes in animals that are relatively reliable, especially when more than one measure of depression or anhedonia is used. 

Review of the literature highlighted more contrast between experimental studies that have employed behavioral paradigms to investigate anxiety-like behavior. In measures of locomotive changes, Elevated Plus Maze (EPM), or Open Field Test (OFT), many studies report identifying deficits or changes indicative of anxiety-like behavior [51,52,53,57,58,59,60,61,62,63,65,67,68,69,75,76,77,78,79]. However, a smaller number of studies identified in review failed to elucidate differences in anxiety-like behavior or locomotion associated with reserpine administration [49,50,51,53,55,64,72,73,85,86,87]. Other distinctive paradigms employed within the literature provide evidence for anxiety-like behaviors in measures of catalepsy [75], burrowing [57], grimace [72,75,81], four-plate test [75], and dark-light box [65]. However, further empirical support is necessary to validate these outcomes. Overall, the current consensus for the reserpine model’s FMS-like anxiogenic properties is unclear due to the segregated nature of the current literature. It is important to note that these studies implemented a variety of experimental designs and timelines, and thus, it is challenging to draw clear conclusions from a body of literature employing a glaring variation in dependent variables, time courses, and statistical procedures. Therefore, we cannot confidently regard the reserpine model as reliable in producing these anxiety-like behaviors, whether this be due to inefficacy of the model itself or the notable experimental differences highlighted in review. Future research should seek to evaluate these results from a meta-analytic approach to draw clearer conclusions about the effects of the reserpine model on animal anxiety-like behavior. 

Investigations into the realms of cognition and sleep associated with the reserpine model are much clearer, given that the identified literature is marginal. All cognitive investigations identified in review utilizing the Morris Water Maze (MWM) paradigm highlighted cognitive deficits associated with the reserpine model [68,69,70,78]. Further cognitive deficits were also identified in measures of learning, such as discrimination or fear conditioning [86], and memory, such as passive avoidance [68,78]. However, there was a failure to highlight cognitive deficits identified within measures of step-down inhibitory avoidance (SDIA), an assessment of aversive memory and learning [86]. All studies identified in review of the literature investigating the impact of reserpine administration on sleep in animals identified significant alterations in sleep patterns, durations, or intensities, implicative of a robust effect of the reserpine model on producing altered sleep quality as is observed in FMS patients [80,82,89]. 

Many treatments were investigated in their ability to alleviate cognitive and affective manifestations in animals. In assessments where FST was the sole measure of affect, the following treatments were reported as effective in alleviating behavioral despair associated with the reserpine model of FMS: curcumin [38], Phα1β [39], laser irradiation [41], duloxetine or a duloxetine-gamma irradiation combination treatment [41], imipramine [43], I2R agonists [43], combination treatment with folic acid and melatonin [44], B1R receptor KO or selective antagonists [45], and Cur-IONPs [46]. In other affective measures of FMS-like pain, evidence has been provided for improvement with a variety of antidepressants, including imipramine [49,54,56], duloxetine [37], desvenlafaxine [37], amitryptiline [59], citalopram [66], vortioxetine [75], and fluoxetine [61,62]. Evidence has also been provided for anticonvulsants gabapentin [61] and analog pregabalin [58]. Additionally, changes to diet and exercise have been shown to alleviate negative emotionality associated with this model [53,72]. Further alternative approaches have been identified for improving affect in animals as well, including ferulic acid [49], resveratrol [52], tactile stimulation [56], fisetin [58], melatonin [22,77], CoQ10 [60], Yukmijihwang-won [61], carotenoids [85], electroacupuncture [22,79], and thymoquinone [66]. Drugs that promote neuro-recovery, such a cerebrolysin [61], and neuroprotection, such as TIQ and 1MeTIQ [50], provide further promise. Additional investigations into the role of underlying biological mechanisms associated with negative affect in reserpine animals have identified treatments geared towards N/OFQ peptide receptor agonists [55], TRPV1 antagonists [59], and organoselenium compounds [54]. Cognitive symptoms associated with the reserpine model were also treated successfully across various studies highlighted in review. Evidence has been provided for gabapentin in alleviating cognitive deficits in animals [68,69,70,76]. Evidence has also been provided for more naturalistic compounds such as imperatorin [68], esculetin [69], daphnetin [70], and Angelica archangelica “angel plant” extract [76]. Additionally, enrichment of an animal’s environment provided alleviation for various affective and cognitive deficits [83]. A large portion of treatments identified for the alleviation of reserpine-induced negative emotionality and cognitive decline were more organic in nature, providing an interesting perspective towards “nonpharmacologic” multidimensional management of FMS-like pain. However, having such a large body of literature with variations in methodologies, experimental designs, and sample sizes yields a mixed evaluation overall of the reserpine model’s ability to replicate key dimensions of the FMS experience.

### 3.2. Acidic Saline

The acidic saline model, developed by Sluka, Kalra and Moore in 2001 [30], was initially established by investigating the nociceptive effect of two unilateral injections of various pH saline into the gastrocnemius muscle. Acid saline comparison procedures consisted of 0.1 mL injections of pH 4.0, pH 5.0, pH 6.0, and pH 7.2, with a second injection of the same volume and acidity occurring 5 days later. A second experiment further investigated the impact of two 4.0 pH saline injections instead occurring 2- or 10-days apart. Male Sprague-Dawley rats (250–400 g) were compared across measures of MPWTs, Thermal Withdrawal Latencies (TWLs), motor function, muscle histology, intramuscular pH, and assessment of peripheral mechanism. Results revealed that initial injection of 4.0, 5.0 and 6.0 pH saline decreased mechanical thresholds in the ipsilateral paw at 4 h following the first injection but were restored to baseline at 24 h. Following the second injection, mechanical thresholds were significantly reduced in both the ipsilateral and contralateral paws among the 4.0 pH saline group with maintenance persisting for 4 weeks compared to all other pH groups. The second injection of 5.0 pH saline also produced significantly reduced withdrawal thresholds in both paws, compared to the 7.2 pH group. Analysis of the effects of time of injection within the 4.0 pH saline group revealed that a second injection occurring on day 2 and day 5 equivocally reduced withdrawal thresholds in both paws, while a second injection administered on day 10 produced no change. Injection of lidocaine into the ipsilateral gastrocnemius 24 h after the second 4.0 pH saline injection ceased plantar sensitization and increased ipsilateral thresholds 10 to 15 min following administration. However, there were no changes seen in the reduced contralateral thresholds. Dorsal rhizotomy revealed comparable results, with no evidence of ipsilateral sensitization 24 h after surgery, yet no recovery was present in the contralateral paw. Administration of various pH saline levels, nor variations in the second injection administration timeline produced changes in TWLs. There were no differences in rota-rod performance or body weight changes between groups. Muscle histology revealed no significant muscle injury across groups further than what might be reported as consistent with insertion of a needle into the muscle. Changes in intramuscular pH were reported as significantly reduced initially among the 4.0, 5.0, and 6.0 pH groups, but were restored to levels of the 7.2 pH group no later than 7 min after saline administration [30]. 

The initial development of this model provided face validity through its ability to produce bilaterally reduced thresholds of a non-inflammatory nature with a lack of significant tissue damage. The mechanisms of action associated with the bilateral hyperalgesia noted in the model through repeated intramuscular injections, two to five days apart, are understood to be related to the activation of muscle afferent acid sensing ion channel (ASIC) 3. However, these peripheral channels are not implicated during the 4-week maintenance of the model [90,91]. Studies investigating the hyperalgesia-associated mechanisms following the second injection provide evidence for increases in excitatory neurotransmitters (i.e., increases in glutamate mediated excitatory activity) and decreases in inhibitory neurotransmitters in the rostral ventromedial medulla (RVM), implicating potentially compounding supraspinal mechanisms for the induction phase of the model [91,92]. In terms of maintenance of this hypersensitivity, evidence for central sensitization has been provided by reported increases in neuronal receptive fields, as well as an increased responsiveness to both painful and non-painful stimuli [90]. During the maintenance phase, there are further implications for persistent hyperalgesia also being associated with increased spinal cord glutamate [91]. Briefly, this model is understood to employ muscle afferent and supraspinal mechanisms during the induction of hyperalgesia, and spinal/supraspinal central mechanisms during the maintenance phase [91]. 

While investigation into the underlying biological mechanisms associated with the development of the model are vital, a well-rounded understanding of the model’s ability to replicate the FMS experience across the affective and cognitive dimensions of pain provides insight into the clinical translatability of studies aimed at the expansion of comprehensive FMS management.

#### Acidic Saline Evaluation

Identified studies that evaluated the affective or cognitive dimensions of pain associated with the acidic saline model are grouped in Table 2. Investigations of the acidic saline model’s ability to induce negative emotionality associated with FMS-like pain yielded mixed results, with negative affect being inconsistent across various behavioral assays. Studies were identified with contrasting results within measures of EPM [93,94,95,96,97], OFT [65,93,97,98], sucrose measures [93,97], and FST [65,93,97,98]. Differences in avoidance behavior were not produced by the administration of acidic saline [65,99].

While investigations into cognitive differences as induced by the acidic saline model show promise, results identified within this review currently provide mixed evidence for cognitive deficits among animals randomized to the FMS-like model. While assessment of Learned Avoidance (LA) failed to highlight cognitive differences among FMS animals [99], more diversified experimental design did find significant cognitive differences related to acidic saline administration [100]. Investigations into sleep reported rather consistent changes in sleep patterns and reactivity among animals administered acidic saline [101,102], although related evidence for sleep fragmentation in contributing to the development of hyperalgesia has been provided as well [103].

While manifestations of negative emotionality varied significantly across studies, the efficacy of various treatment mechanisms for the alleviation of negative affect was investigated in the case where negative affect was able to be effectively identified. Diazepam, pregabalin, and duloxetine were inconsistent in their ability to relieve negative affect within animals, dependent on the behavioral paradigm that was being investigated [93,94]. Mirogabalin, however, did show promise in alleviating the negative affect observed in animals in measures of EPM and OFT [95]. Evidence was also identified for electroacupuncture in the management of FMS-like negative affect [98]. The only effective treatment identified within this review for the alleviation of cognitive deficits was mirogabalin [100]. Although the results for the model’s ability to induce the negative affect associated with FMS clinical diagnosis provided little clarity, there is much more promise for the model’s ability to induce cognitive deficits and sleep disturbances often reported by FMS patients. However, this body of literature investigating cognitive deficits and sleep is limited. Therefore, future research should seek to add to these bodies of literature, as little work has been done in replicating these deficits or investigating therapeutic approaches for alleviating cognitive decline and sleep disturbances in animals.

### 3.3. Fatigue-Enhanced Muscle Pain

Closely related to the acidic saline model, is the fatigue-enhanced muscle pain model. Within this model, animals are subjected to muscle fatigue by a wheel running activity or direct muscle stimulation. Fatigue protocol is followed by muscular insult with 0.03% carrageenan or two injections of 5.0 pH saline, 5 days apart [60]. The original development of this model [31] investigated variations in pH injections (4.0, 5.0, 6.0, or 7.2) within male mice (C57BL/6J, 19–22 g), and found reduced thresholds following 5.0 pH injections. No significant differences in measures of muscle damage or composition were identified, despite animals showing a significant decrease in grip force [31].

The reduced muscle force, lack of inflammation, and maintenance of muscle composition provide important insight into the peripheral mechanisms, or perhaps lack thereof, of this model [31,104]. However, important central mechanisms have been identified by subsequent research as well. The rostroventral medulla (RVM) has been implied as a crucial cortical area for the effects of this model, with blockade of NMDA receptors, specifically the p-NR1 subunit, preventing the development of hyperalgesia [104,105,106]. However, there are further implications for sex differences, with female mice showing greater magnitudes of hyperalgesia that are not affected by ovariectomy [32,104]. Overall, the mechanisms underlying the effects of the model are not entirely clear [91].

#### Evaluation of Fatigue-Enhanced Muscle Pain Model

Review yielded a single study that investigated the affective or cognitive components associated with the fatigue-enhanced muscle pain model [99] (Table 3). However, this study highlighted a failure on behalf of the model to produce negative affectivity or cognitive deficits, as assessed in escape-avoidance and learned avoidance behavior. There is a great need for future research into the cognitive and affective behavioral components, as well as prospective treatment approaches for this model. 

### 3.4. Subchronic Swim Stress

The subchronic swim stress model was developed by Quintero et al. [36], under the hypotheses that short and emotionally non-noxious stress may produce hyperalgesia by mechanisms of involvement of serotonergic systems. This stress protocol consisted of forcing male rats (Sprague-Dawley, 150–300 g) to swim in a cylinder tube, similar to FST analyses, for 10 min on day 1 and 20 min on days 2 and 3. Involvement of serotonergic systems were explored by pretreating animals with tryptophan (3 mg/kg i.p.) and Selective Serotonin Reuptake Inhibitors (SSRIs), including clomipramine (2.5 mg/kg, i.p.) and fluoxetine (0.25 mg/kg, i.p.). By the third swimming session, vehicle control treated animals displayed significantly more immobility behavior compared to clomipramine and fluoxetine treated animals. TWLs were significantly reduced in animals who underwent the subchronic swim stress, and these latencies were significantly improved in animals treated with clomipramine, fluoxetine, and tryptophan. Formalin test scores revealed that animals who underwent the forced swim protocol had significantly higher pain scores in the interphase and the late phase than those who experienced the sham swimming condition. Fluoxetine, clomipramine, and tryptophan attenuated these scores. The authors concluded that this model produces long lasting pain sensitivity by diminishing central nervous system activity. However, an interesting note about the approach of this model is that FST is a measure of depressive-like behavior in animals. This would imply that repeated measures of FST within experimental designs may not be observing organic pain as a result of experimental manipulation, but rather, that there is a potential for these studies to be inducing secondary hyperalgesia through prolonged repeated measures of FST analyses. 

While results from Quintero et al. [36] imply that the mechanisms of action associated with this model are serotonergic, further research into the underlying biological components responsible for the behavioral expressions of this model have yielded results primarily rooted in central processing. In the event of formalin insult following the induction of the model, rats had significantly higher levels of c-Fos-immunoreactive nuclei in the ipsilateral and contralateral lumbar dorsal horn (L4, L5) [107,108,109]. Animals not subjected to nociceptive stimuli showed inconsistent patterns of c-Fos expression, however, there were higher numbers of faintly c-Fos positive nuclei in animals subjected to forced swim [108]. Inflammatory insult by carrageenan paired with subchronic swim also induced further impairments, which were effectively blocked by preventative treatment with SSRIs [110], further supporting the notion of serotonergic mechanisms. Suarez-Roca et al. [109] and Quintero et al. [107] also provided evidence for GABAergic system deficits during the formalin test, with subchronic swim animals showing decreased concentrations of GABA from basal periods into the interphase period of the test. Further investigations into central mechanisms associated with subchronic swim highlighted the potential role of μ-opioid and NMDA receptors in the development of hyperalgesia, and the role of NMDA receptors in maintenance [107,109]. 

While understanding the biological contributions towards developing effective preclinical FMS models is crucial, it is just as vital to understand the model’s ability to identify and replicate concomitant affective and cognitive experiences. Many articles identified in review primarily assessed measures of depression-like behavior using the FST. It is challenging to assess the implications of behavioral results using the same methodology as was used to induce hyperalgesia, such as evaluating differences in immobility time across each session of forced swim. Identified studies that assessed differences in forced swim behavior while inducing hyperalgesia through forced swim sessions are excluded from Table 4 and Table A1, but are discussed based on their key findings related to FST variables.

#### 3.4.1. Forced Swim Test Analyses

Okamoto et al. [122] evaluated the combination of high and low estrogen status alongside subchronic swim induced stress on TMJ-evoked spinal activity in female rats. Immobility time was increased among both high estrogen and low estrogen rats by day 3 of conditioning, suggesting no interactions between estrogen and development of the model. Cao et al. [123] investigated underlying mechanisms associated with visceral hypersensitivity in female rats subjected to subchronic swim stress. Repeated swims were associated with significant increases in immobility time across each of the 3 days. Ji et al. [124] hypothesized pronociceptive effects of estradiol and antinociceptive effects of testosterone within the subchronic swim stress paradigm. Immobility time in the first 3 min of the swimming paradigm was significantly decreased among female rats compared to male rats in the first 2 days. Nakatani et al. [125] provided evidence for immobility time being significantly increased among male rats on day 1 of the subchronic swim paradigm, with fluoxetine (10 mg/kg) offering protective effects against this increased immobility. Nakatani et al. [126] noted a difference between groups among animals subjected to subchronic swim, with animals being treated with ethanol or sake displaying less immobility on the final day of the swim paradigm compared to controls. Notably, these studies are important for understanding the behavioral nuances while hyperalgesia is being induced. However, they do not provide sufficient evidence for the robust affective or cognitive impacts that may be associated with the induction of hypersensitivity. 

#### 3.4.2. Subchronic Swim Evaluation

Studies identified within this review that evaluated the affective or cognitive dimensions of pain associated with the swim stress model of FMS-like pain can be found in Table 4. Within measures of affectivity, data yielded mixed results for the assessment of the negative emotionality within this model, dependent on the behavioral paradigm employed. Disregarding studies with sole assessment of immobility time during stress acquisition and including measures of immobility or FST alongside other behavioral assessments, results are consistent in that the swim stress model produces increased immobility behavior either over time or in a single FST assessment [112,113,115,116,117,120]. There have been consistent results identifying the presence of anxiety-like behavior associated with the model in mirror-chamber assessments as well [115,117,120]. Among more commonly investigated anxiety-like behaviors, there are mixed results in measures of EPM, with prominence leading towards anxiety-like behaviors not being consistently found within the model (significance identified: [114,115]; significance not identified: [111,113,116,117,120]). In measures of OFT, results have repeatedly implied that the model does not produce anxiety-like changes in locomotion [116,118,119]. For measures of depression-like behavior, SPT and TST results have consistently suggested negative emotionality in animals associated with swim stress [112,113], with a noted prevalence among females that signals towards sexual dimorphisms [127]. The only study highlighted in review that reported an absence of depression-like behaviors may be a result of a stark difference in experimental design, wherein the cumulative impact of inflammatory pain and swim stress was investigated [121]. However, alterations in motivational elements were also identified in review, such as increased escape behavior [113] or changes in general locomotion [111,118,119]. 

Only one study was identified investigating the cognitive-evaluative processes associated with the swim stress model, with deficits being reported among chronic swim animals in a measure of passive avoidance learning [118,119]. Furthermore, no studies were identified evaluating the impact of subchronic swim stress on sleep. Preclinical investigations should seek to further explore the use of this model within these valuable realms of the FMS experience. 

Within the identified research into negative affectivity and cognition, various treatments were reported as significantly attenuating negative effects within the affective or cognitive pain dimensions. Venlafaxine, epigallocatechin gallate (green tea), fluoxetine, imipramine, ifenprodil, and morphine were selectively effective in alleviating reported negative affectivity [111,112,115,117,120]. The one identified investigation into learning deficits identified two potential treatments for alleviation of cognitive deficit: L-NAME and L-Arginine, with L-NAME producing stronger results comparatively [111,112,115,117,118,119,120]. Investigations into L-NAME reported an attenuation of changes in locomotion associated with OFT. However, the changes in exploratory behavior reported as a result of subchronic swim stress were not reliably generalizable to anxiety-like behavior, thus, making it difficult to draw conclusions about the effectiveness of L-NAME in improving negative affect. Future research should seek to clearly evaluate if treatment approaches reported as beneficial within affective dimensions of the pain experience translate to the cognitive realm, and vice versa.

### 3.5. Cold Stress

Original evidence for cold stress contributing to the development of nociception was displayed by Kita et al. [128]. Further variations in this methodology has been explored [129], but for the scope of this review, we comparatively assess intermittent cold stress (ICS) methodology as discussed by Nasu, Taguchi, and Mizumura [130], who had a goal of developing a single stress-type based model of chronic muscle hyperalgesia. To evaluate the potential impact of cold stress, male rats (Sprague-Dawley, 200 g) were randomized to a cold stress condition of 4 °C/39.2 °F, −3 °C/26.6 °F, or a control condition wherein animals were exposed to the same chambers but experienced no variation in temperature. The procedure consisted of overnight exposure to their randomized temperature group for 15 h (19:00–10:00), before alternating 30 min exposures to room temperature (22 °C/71.6 °F) and the randomized cold temperature for 7.5 h (10:00–17:30). This exposure procedure persisted for 5 days. Mechanical thresholds were significantly reduced for animals exposed to cold stress, with the −3 °C group displaying persistent bilateral hyperalgesia for up to 42 days after final exposure to the stress procedure. 

Currently understood biological mechanisms associated with this model include evidence for alterations in 5-HT synthesis, as significant supraspinal reductions in 5-HT and its metabolites have been observed in the hypothalamus, midbrain, thalamus, pons, and medulla oblongata [131]. Evidence has also been provided for hyposensitivity in supraspinal μ-opioid receptors [132]. Further central mechanism contributions have been identified in the alleviation of cold stress hyperalgesia by blocking substance P, CGRP, and NMDA receptors [133,134,135], as well as through post-translational modification of proteins related to neurotransmitter release and axon elongation or plasticity [136]. While various identified contributing mechanisms to cold stress have failed to identify a consistent underlying mechanism responsible for the reported hyperalgesia, consistent evidence has been provided for alterations in central nervous system factors. Although these underlying biological contributions are important to understanding overall pathology of the model, elucidating the multidimensionality of cold-stress related pain is vital for translatability to an FMS population.

#### Cold Stress Evaluation

Review of the literature for the intermittent cold-stress model of FMS-like pain in animals identified 4 studies that investigated associated alterations in affect (Table 5). Although the current literature is limited, current data supports the model’s ability to induce depression-like behaviors in animals as has been measured in TST [137], the evasion test [138], and FST [139]. However, one study did fail to find significant differences is TST behavior [33]. Two studies incorporated investigations into anxiety-like behavior, and only one identified anxiety-like effects associated with the cold stress model [33,138]. Due to this body of literature being limited, it is crucial to conduct additional studies incorporating anxiety-like behavioral paradigms to determine if this model is truly ineffective in producing these behavioral deficits.

Within these limited studies, treatments for significantly improving depression-like behaviors in animals produced by the cold stress model have been identified [137,138,139]. Gabapentin was identified as having improved evasion behaviors, implicative of improvement in depression-like behaviors, alongside improvement in evoked pain measures within animals subjected to the cold stress model [138]. *Valeriana fauriei* (VF) extract also produced significant improvement in evoked pain and depression-like behaviors [137]. Imipramine and Neurotropin were also reported as having improved depression-like behaviors in cold-stressed animals [139], but further research must be conducted to evaluate if these effects are also present within the sensory dimension of pain. Review of the literature identified a significant research gap, with no investigations having been conducted highlighting the effect of the ICS model on cognition or sleep. Future research should make effort to incorporate these measures, as well as validate the pharmacological profile associated with them, in order to create a more robust multidimensional understanding of the implications of the cold stress model of FMS-like animal pain.

### 3.6. Sound Stress

Studies on the impact of the exposure to sound as a stressor have progressed dramatically from their initial introductions [140] to more recent methodology being produced in relation to its impact on nociceptive functioning. Khasar, Green, and Levine [35] performed primary investigations into this methodology’s integration, wherein male Sprague-Dawley rats (250–380 g) were exposed to a 5 or 10 s 105 dB tone of mixed frequencies (11–19 kHz), every minute at random times, for a period of 30 min on days 1, 3, and 4. This protocol, paired with an inflammatory bradykinin insult, revealed a cumulative effect in reducing mechanical thresholds. Investigation into biological mechanisms suggested a sympathetic-independent alteration that increased epinephrine release from the adrenal medulla. 

Further investigations into the mechanisms associated with the development of FMS-like pain through sound stress exposure suggest a combination of central and peripheral mechanisms [91]. Peripherally, epinephrine and catecholamine synthesizing enzymes are critical for maintenance of hyperalgesia, alongside other inflammatory peripheral mechanisms [34,35,141,142]. There is also evidence for elusive underlying central mechanisms, as displayed by secondary visceral, paw, and temporomandibular hyperalgesia [141,143]. 

#### Sound Stress Evaluation 

Review procedures identified 4 experimental studies investigating the affective or cognitive dimensions associated with the sound stress model in animals (Table 6). While this body of literature is still limited, there is progress in determining the model’s ability to replicate the negative emotionality commonly observed in manifestations of FMS pain. Of the work done investigating this negative emotionality, the majority of studies imply an ability of the model to produce change in anxiety-like behavior among animals, as has been observed in measures of EPM [143,144], OFT [144,145,146], and grimace scale [146]. However, contrasting results evaluating a change in behavior in measures of EPM were presented as well [145]. We note that sample sizes and timepoint of data collection vary across studies, and thus, further research is warranted regarding the conditions within which anxiety-like behaviors are associated with the sound stress model. Future research should additionally be geared towards the incorporation of depression-like, sleep, and cognitive-evaluative behaviors in relation to the sound stress model.

Of the studies that identified this negative affectivity within animals, two treatment methodologies were identified as having provided significant improvement: riparin III [145] and CGRP antagonist, olcegepant [146]. Data presented identified these treatments as effective in altering behaviors as measured in OFT [145,146] and spontaneous pain-associated grimace behaviors [146]. These promising treatment mechanisms should be further investigated within additional parameters of negative emotionality, cognitive deficits, and sleep disturbances.

## 4. Discussion

According to current preclinical approaches, a favorable animal model of FMS should possess the ability to produce the commonly reported FMS symptoms and comorbidities through a prominent pathological mechanism, while simultaneously replicating outcomes of primary disorder management profiles [91,141]. While the scope of this review was not to assess prominent pathological mechanisms, we did seek to identify the favorability of preclinical FMS models in their ability to replicate the affective and cognitive dimensions of the pain experience. Additionally, we sought to highlight evidence for currently established management profiles, as well as novel potential treatment methodologies for their capability of offering improvement in these pain realms. While this review did not serve to label “good” and “bad” models of FMS-like pain, as each of these models have subjective strengths and weaknesses, we do seek to offer transparent evaluation of common FMS manifestations that often go overlooked, and therefore serve to possibly dampen translatability for developing robust FMS treatment approaches.

Within the goal of offering transparent evaluation of each of these preclinical models, the data for affective and cognitive replicability yield uncertainty, especially among the widely embraced models of FMS-like pain. In the use of the reserpine model, there is empirical support for the model producing the negative sensory experience as would be expected in coining it as a model of pain. Furthermore, there is substantial evidence for the model in producing depression-like behaviors among animals, especially when more than one measure of depression or anhedonia is incorporated into experimental measures. Although there are only a few studies, there is also evidence for the model producing changes in sleep as is reported by FMS patients. However, there is discord regarding the model’s ability to produce anxiety-like behaviors. Overall, a handful of studies were identified that substantiated the reserpine model’s ability to produce FMS-like manifestations across the sensory, affective, and cognitive realms [68,69,70,76]. Within these studies, evidence for the novel use of coumarins, angel plant extract, and anticonvulsant gabapentin, were highlighted for attenuating the nociceptive effects of reserpine across all three pain dimensions [68,69,70,76].

Literature surrounding the acidic saline model provides consistent support for the model’s ability to produce a painful sensory experience. Further, the identified studies yielded results implicative of negative impacts on sleep as a result of acidic saline insult. However, the literature identified in the review produced obscurity for the model’s ability to replicate negative affect within various measures of avoidance and depression- or anxiety-like behaviors. While there was only one study that allowed for a clear evaluation of cognitive deficits, there is promise in the data provided given that differences in behavior across various paradigms may be interpreted as alterations in cognition, affect, and sensation [100]. Within this study, the only treatment investigated, mirogabalin, offered alleviation in measures of cognition, and potentially measures of affect, but no improvement in pain thresholds.

Evaluation of the subchronic swim stress model literature identified a rather consistent impact of the model in inducing sensory pain behavior. The identified literature also favors the notion of a reliable induction of depression-like behaviors among animals. However, the evidence for the presence of anxiety-like behaviors was mixed, leaving assessment of the model’s ability to induce predictable negative affectivity unclear. A primary example of this lies within the only identified study to investigate all three pain dimensions associated with subchronic swim stress, where evoked pain behaviors were reported, negative impacts were identified in avoidance learning, but no changes were highlighted in measures of anxiety-like behavior [118,119]. However, due to the lack of conclusive evidence for negative affectivity, there was no treatment identified that offered alleviation across the affective, cognitive, and sensory pain dimensions. 

Only one study was identified as having evaluated affect and cognition in the fatigue-enhanced muscle pain model [96]. Within this study, there was no evidence for differences in measures of affect or cognition as a result of fatigue and muscle insult protocol. However, additional limitations of this study arise in that neither reflexive pain nor potential treatments were investigated [99]. Similarly, no studies were identified that evaluated the cold-stress model or the sound-stress model from an affective, cognitive, and sensory perspective, thus limiting the ability to evaluate treatment methodologies within these models. In addition to a need for supplementary multidimensional validations of these models, there is significant gap to be filled by investigations of sleep disturbances associated with each, as no studies analyzing sleep changes were identified in the current review.

The primary issue highlighted by review of the literature is an absence of studies that evaluate the FMS-like pain experience, or its prospective management methodologies, from a multidimensional perspective. Most experimental designs employed incorporate exploration into the affective experience of pain. However, cognitive dimensions are reported among patients as equally, if not more, debilitating than physical pain and affect [19]. There is great value in evaluating the affective and cognitive dimensions as separate contributors to the same pain experience, particularly in FMS, due to evidence presented that mood disturbances themselves may not mediate the observed “fibro fog” among patients [19]. Due to the significant absence of these multidimensional approaches in preclinical literature, there is a disconnect between current clinical approaches focused on multidimensional treatment. It is of necessity that future investigations utilizing preclinical FMS models incorporate behavioral paradigms from each the affective, cognitive, and sensory dimensions of pain in order to improve understanding of both the face validity of the model, and to offer a robust evaluation of prospective treatments.

There is also substantial research left to be done individually within many of these models. The literature identified for cold-stress, sound-stress, and fatigue-enhanced muscle pain models of FMS-like pain present very few studies that have evaluated the impact of these models beyond the sensory experience. As presented by Melzack and Casey in 1968 [3], evaluation of pain from solely a sensory-discriminative standpoint does not serve to improve the pain experience as a whole. However, despite the major gap in the literature, there is promise to be potentially fulfilled by these models in representing preclinical FMS pain. Particularly within the identified sound-stress and cold-stress literature, there is evidence for the presence of negative emotionality associated with each, despite there not being a clear consensus on overall effectiveness for replication. Given this, it is vital that more affective and cognitive behavioral investigations be incorporated into future experimental designs.

With the approach employed, there are limitations to be noted within the scope of this review. Primarily, without a statistical or meta-analytic approach to the outcomes of each identified study, there is significant subjectivity in evaluating the current consensus of the literature for each model’s ability to replicate the affective and cognitive dimensions of FMS pain. This point is emphasized given that results from the identified studies varied significantly due to variation in sample sizes, experimental timelines, and specific behavioral paradigms employed. Furthermore, we cannot say that every behavioral paradigm within the realms of affect or cognition are equivocal in their assessments of underlying pain mechanisms or experiences. More succinctly, the underlying affective experience as assessed in EPM, may not be equivalent to the affective experience as is assessed in OFT or avoidance paradigms. The same can be said for measures of cognition. Therefore, we encourage future researchers to incorporate a variety of behavioral paradigms into their experimental designs, given that evidence of negative emotionality may be deduced in some behavioral paradigms and not in others. Additionally, we note that future work should expound upon the current state of this review, in the form of investigating more databases and incorporating more evidence within the sensory realm of each preclinical model. However, this review poses significance in that it is the first to place emphasis on the affective and cognitive manifestations of preclinical FMS models. Previous reviews have been conducted investigating the underlying biological mechanisms associated with each of these FMS-like models, with some discussion of their affective and cognitive evidence in the nature of face validity [91,141]. However, an animal model of FMS must replicate the commonly reported symptoms and comorbidities of FMS, which now include the foundational diagnostic components of affective and cognitive deficits [15].

Overall, substantial gaps in the literature must be filled before conclusive assessments can be made about the effectiveness of any of the discussed models as a representation of FMS pain. It is inefficient to hope for translational efficacy if preclinical standards do not adhere to clinical diagnostics when trying to bridge the gap from preclinical findings to clinical application. Clinical manifestations of idiopathic pain are communicated through the intricate relationship of the sensory-discriminative, affective-motivational, and cognitive-evaluative dimensions of pain. This is not to say all preclinical investigations, by necessity, should fit the exact framework of these clinical manifestations, but rather, attempts to inform the clinical realm translationally might be more robust if preclinical research uses its advantages to attempt to better understand the multidimensionality of the pain experience being reported. When attempting to translate preclinical work into the clinical idiopathic pain realm, it is important that we are using models that provide validity for the multiple facets of the disorder, especially when its definitive characteristics span across a multitude of the pain dimensions. When future preclinical research employs this approach, exponential growth will be made towards the understanding and management of idiopathic disorders, such as FMS, across each realm of the multifaceted pain experience.

## 5. Conclusions

Review of the current literature for preclinical FMS-like pain yielded mixed results for whether these models offer affective and cognitive face validity. However, each model possesses strengths and weaknesses in translating the affective and cognitive deficits experienced by many FMS patients. Several treatments were identified as effective in attenuating affective and cognitive deficits; however, most of these treatments were pharmacological, which does not serve to represent the multidisciplinary approaches primarily employed for FMS management. Future studies should aim to better validate the multidimensional replicability of these models, as well as further investigate preclinical treatments across the affective, cognitive, and sensory dimensions of pain to maximize translational efficacy.

## Figures and Tables

**Table 1:** Summary of articles identified utilizing the reserpine model. Articles are grouped by their investigation of affect and cognition, as well as by treatments investigated for the alleviation of affective and cognitive deficits.

	Affective	Cognitive	Treatment for Affect/Cognition
Reserpine			
Significant:	[22,37,38,39,40,41,42,43,44,45,46,47,48,49,50,51,52,53,54,55,56,57,58,59,60,61,62,63,64,65,66,67,68,69,70,72,73,74,75,76,77,78,79,80,81,82]	[68,69,70,76,83]	Antidepressants: [37,42,43,47,49,54,56,59,61,62,66,78,80,81] Parkinson’s DA agonist: [64,78] Anticonvulsants: [55,58,61,68,69,70,72,76,81] Neurorecovery/neuroprotective: [47,50,62] Organics: [38,44,49,52,58,60,61,63,66,68,69,70,74,75,76] Environment/Diet/Exercise: [53,56,72,83] Alternative Measures: [22,41,42,84] Other: [43,45,47,54,55,57,59,61]
Non-significant:	[83,85]	-	Anticonvulsants: [39,45,52,57,63,65] NSAID: [39,63,81] Opioid Partial Agonist: [81] Organics: [39,46,52] Alternative Measures: [22] Other: [22,43,51,57,85]
Inconclusive:	[80,86,87,88]	[80,88]	Benzodiazepines: [88] Organics: [86,87] Other: [88]
			Not Investigated: Antidepressants: [40] Anticonvulsants: [59] Other: [57,61,85,88]

**Table 2:** Summary of articles identified utilizing the acidic saline model. Articles are grouped by their investigation of affect and cognition, as well as by treatments investigated for the alleviation of affective and cognitive deficits.

	Affective	Cognitive	Sleep	Treatment for Affect/Cognition
Acidic Saline				
Significant:	[65,93,94,95,96,97]	[100]	[101,102,103]	Antidepressants: [97] Benzodiazepines: [97] Anticonvulsants: [65,93,94,97,99] Alternative Devices: [98] Organics: [93] Other: [93,94,95,96,97,99]
Non-significant:	[96]	[99]	-	Organics: [94]
Inconclusive:	-	[94]	-	-

**Table 3:** Summary of articles identified utilizing the fatigue-enhanced muscle pain model. Articles are grouped by their investigation of affect and cognition, as well as by treatments investigated for the alleviation of affective and cognitive deficits.

	Affective	Cognitive	Treatment for Affect/Cognition
Fatigue-Enhanced Muscle Pain			
Non-significant:	[99]	[99]	-

**Table 4:** Summary of articles identified utilizing the subchronic swim stress model. Articles are grouped by their investigation of affect and cognition, as well as by treatments investigated for the alleviation of affective and cognitive deficits.

	Affective	Cognitive	Treatment for Affect/Cognition
Subchronic Swim Stress			
Significant:	[111,112,113,114,115,116,117]	[118,119]	Antidepressants: [113,117,120] Narcotic analgesic: [111] NMDA receptor antagonist: [113] NOS antagonist: [118,119] Organics: [115,117,118,119]
Non-significant:	[118,119]	-	-
Inconclusive:	[120,121]	-	-

**Table 5:** Summary of articles identified utilizing the cold stress model. Articles are grouped by their investigation of affect and cognition, as well as by treatments investigated for the alleviation of affective and cognitive deficits.

	Affective	Cognitive	Treatment for Affect/Cognition
Cold Stress			
Significant:	[137,138,139]	-	Antidepressants: [139] Anticonvulsants: [138] Non-protein extract: [139] Organics: [137]
Non-significant:	[129]	-	-
			Not investigated: Antidepressants: [129]

**Table 6:** Summary of articles identified utilizing the sound stress model. Articles are grouped by their investigation of affect and cognition, as well as by treatments investigated for the alleviation of affective and cognitive deficits.

	Affective	Treatment for Affect/Cognition
Sound Stress		
Significant:	[143,144,146]	CGRP antagonist: [146] Alkamide-type alkaloid: [145]
Inconclusive:	[145]	-
		Not investigated: Anticonvulsants: [144] Narcotic analgesics: [144] NSAIDs: [144]

## Data Availability

No new data were created or analyzed in this study. Data sharing is not applicable to this article.

## Abbreviations

FDA: Food and Drug Administration; FMS, fibromyalgia; ACR, American College of Rheumatology; NSAIDs, non-steroidal anti-inflammatory drugs; EULAR, European Alliance of Associations for Rheumatology; CBT, cognitive-behavioral therapy; WPI, Widespread Pain Index; SS, Symptom Severity; MWT, muscle withdrawal threshold; MPWT, mechanical paw withdrawal threshold; FST, forced swim test; p.o., per oral; s.c., subcutaneous; DA, dopamine; 5-HT, serotonin; NE, norepinephrine; CAN, central nervous system; I2R, imidazoline I2 receptor; B1R, bradykinin B1 receptor; KO, knockout; Cur-IONPs, curcumin-coated iron oxide nanoparticles; TST, tail suspension test; SPT, sucrose preference test; NSFT, novelty suppressed feeding test; EPM, elevated plus maze; OFT, open field test; MWM, Morris water maze; SDIA, step-down inhibitory avoidance; TIQ, tetrahydroisoquinoline; 1MeTIQ, 1-Methyl-1,2,3,4-tetrahydroisoquinoline; N/OFQ, nociception/orphanin FQ peptide; TRPV1, transient receptor potential vanilloid-1; TWL, thermal withdrawal latency; ASIC, acid sensing ion channel; RVM, rostroventral medulla; LA, learned avoidance; NMDA, N-methyl-D-aspartate; SSRI, selective serotonin reuptake inhibitor; GABA, gamma-aminobutyric acid; TMJ, temporomandibular joint; L-NAME, N(G)-Nitro-L-arginine methyl ester; ICS, intermittent cold stress; CGRP, calcitonin-related gene peptide; VF, *Valeriana fauriei*.

## Appendix A

**Table A1:** Summary of studies included in review with note of significance reported for each discussed dependent measure. Implications for evidence of efficacy in replicating FM-like pain within the affective and cognitive dimensions are highlighted, alongside treatments identified as effective in treating affective and cognitive pain behaviors. * reported significance; ✓ reported non-signifiance; # results inconclusive; - not included.

Reference	Behavioral Measures	Affective	Cognitive	Treatment for Affect/Cognition
Reserpine				
Arora et al. (2011) [38]	MPWT * Tail-flick latency * FST *	*	-	Curcumin *
Hubner de Souza et al. (2014) [39]	MPWT * TWL * FST *	*	-	Pha1b ✓ Pregabalin ✓ Diclofenac ✓
Tamano et al. (2016) [40]	MPWT * FST *	*	-	Duloxetine - Milnacipran -
Mohammed (2016) [41]	FST *	*	-	Laser irradiation *
Shibrya et al. (2017) [42]	FST *	*	-	Laser irradiation * Duloxetine *
Moghazy et al. (2017) [47]	FST *	*	-	Cerebrolysin * Citalopram * ATP *
Siemian et al. (2019) [43]	MPWT * FST *	*	-	I2R agonists (2-BFI ✓, phenyzoline ✓, CR4056 *) Imipramine *
Fusco et al. (2019) [44]	MPWT * TWL * Tail-flick latency * FST *	*	-	Melatonin * Folic acid * Melatonin + folic acid *
Dagnino et al. (2020) [45]	MPWT * FST *	*	-	B1R Knockout * B1R antagonist R-715 * Pregabalin ✓
Miyahara et al. (2021) [48]	TWL * FST *	*	-	-
Khadrawy et al. (2021) [46]	FST *	*	-	Cur-IONPs ✓
Ogino et al. (2013) [85]	MPT * Locomotor activity ✓	✓	-	5-HT2C agonists (YM348 ✓, lorcaserin ✓, vabicaserin ✓) 5-HT1A and 5-HT2A agonists (buspirone -, TCB2 -)
Xu et al. (2013) [49]	MPWT * Tail-flick latency * TST * FST * Locomotor activity ✓	*	-	Ferulic acid * Imipramine *
Antkiewicz-Michaluk et al. (2014) [50]	FST * Locomotor activity *	*	-	TIQ * 1MeTIQ *
Klein et al. (2014) [51]	MPWT * TWL * OFT * TST * FST *	*	-	RvD1 ✓ RvD2 ✓ AT-RvD1 ✓
Blasco-Serra et al. (2015) [73]	MPWT * MPT * NSFT * Locomotor activity ✓	*	-	-
Wu et al. (2015) [79]	PWL * EZM ✓ OFT *	*	-	Electroacupuncture *
Klein et al. (2016) [52]	MPWT * TWL * OFT * FST *	*	-	Resveratrol * Rice oil ✓ Resveratrol + rice oil * Pregabalin ✓
Oliveira et al. (2016) [53]	MPWT * TWL * OFT # FST * NSFT * Splash test #	*	-	OMePhSe2 supplemented diet *
Khadrawy et al. (2017) [87]	OFT #	#	-	THC #
Wu et al. (2017) [22]	MPWT * OFT * EZM *	*	-	Electroacupuncture (EA) * 5-HT resynthesis inhibitor pCPA ✓ EA + pCPA ✓
Favero et al. (2017) [75]	Locomotor activity *	*	-	Melatonin *
Blasco-Serra et al. (2017) [37]	NSFT *	*	-	Duloxetine * Desvenlafaxine *
Sousa et al. (2018) [54]	TWL * FST * OFT *	*	-	α- (phenylselanyl) acetophenone * Imipramine *
Nagakura et al. (2018) [77]	MPWT * Locomotor activity * Catalepsy *	*	-	-
Dagnino et al. (2019) [55]	MPWT * TWL * FST * EPM ✓ Rota-rod * Grip strength * Inverted screen *	*	-	NOPr agonist N/OFQ * Selective peptide NOPr antagonist UFP-101 * Pregabalin *
Favero et al. (2019) [74]	Locomotor activity *	*	-	Melatonin *
Roversi et al. (2019) [56]	FST * Splash test * SPT *	*	-	Tactile stimulus * Imipramine *
Brusco et al. (2019) [57]	MPWT * Acetone * Overt nociception * Burrowing * Thigmotaxis * FST *	*	-	B1R Knockout - B2R Knockout - B1R antagonist (DALBk *, SSR240612 ✓) B2R antagonist (Icatibant ✓, FR173657 ✓) Pregabalin ✓
Nagakura et al. (2019) [81]	MPWT * Grimace *	*	-	Gabapentin * Duloxetine * Diclofenac ✓ Buprenorphine ✓ Diazepam ✓
Yao et al. (2020) [58]	MPWT * TWL * Tail-flick latency* RotaRod * FST * OFT * TST *	*	-	Fisetin * Pregabalin *
Tanei et al. (2020) [82]	Grimace *	*	-	-
Fischer et al. (2020) [59]	MPWT * TWL * Tail-flick latency * Muscle strength * Capsaicin * Thigmotaxis * FST *	*	-	TRPV1 antagonists (α-spinasterol *, SB-366791 *) Pregabalin - Amitriptyline *
Brum et al. (2020) [60]	MPWT * Acetone * Grip strength * FST* Catalepsy ✓ Locomotor activity ✓ Thigmotaxis *	*	-	CoQ10 *
Kang et al. (2020) [61]	MPWT * RotaRod FST * OFT *	*	-	Yukmijihwang-won (YJ-01 *, YJ-06 *) Gabapentin * Fluoxetine * Fluoxetine + YJ-01 * Fluoxetine + YJ-06 * 2,2,2-tribromoethanol -
El-Marasy et al. (2021) [62]	OFT * FST *	*	-	Cerebrolysin * Fluoxetine *
Salat & Furgala-Wojas (2021) [78]	MPWT * TWL ✓ FST * Four-plate test * Locomotor activity * Grip strength ✓	*	-	Vortioxetine * Ropinirole *
Mendes et al. (2021) [63]	MPWT * TWL * SPT * Locomotor activity * FST *	*	-	Pha1b * Pregabalin ✓ Diclofenac ✓
Ferrarini et al. (2021) [72]	MPWT * Acetone * Grimace * TST ✓ OFT ✓	*	-	Strength exercise * Aerobic exercise * Pregabalin *
Elkholy et al. (2021) [86]	TWL * OFT #	#	-	Encapsulated cationic liposome (beta-carotene #, lutein #)
Martins et al. (2022) [64]	MPWT * Tail-flick latency * FST * Splash test ✓ EPM ✓ OFT ✓	*	-	Pramipexole *
Álvarez-Pérez et al. (2022) [65]	MPWT * TWL * OFT * Dark/light box * FST *	*	-	Pregabalin -
Kuzay et al. (2022) [66]	FST * TST * NSFT * SPT *	*	-	Citalopram * Thymoquinone (TQ) * Citalopram + TQ *
Zhao et al. (2022) [67]	MPWT * TWL * SPT * OFT * FST *	*	-	-
Souza et al. (2013) [83]	Olfactory fear conditioning * Step-down inhibitory avoidance * Olfactory discrimination ✓ OFT ✓ EPM ✓	✓	*	Environmental enrichment *
Kaur et al. (2019) [68]	MPWT * PAM * Inclined plane * FST * OFT * MWM * Passive avoidance * EPM *	*	*	Gabapentin * Imperatorin *
Singh et al. (2020) [69]	MPWT * PAM * OFT * FST * MWM *	*	*	Gabapentin * Esculetin *
Kaur et al. (2020) [76]	MPWT * PAM * Inclined plane * OFT * MWM * Passive avoidance *	*	*	Angelica archangelica * Gabapentin *
Singh et al. (2021) [70]	MPWT * PAM * FST * MWM *	*	*	Gabapentin * Daphnetin *
Hernandez-Leon et al. (2019) [80]	MPT * MPWT * Acetone * Sleep (W *, SWS-I *, SWS-II *, REM *)	#	#	Fluoxetine *
Murai et al. (2019) [88]	MPT * Sleep (NREM #, REM #, sleep interruptions #)	#	#	GABAb receptor positive allosteric modulator (ASP8062 #) Baclofen # GABAb antagonist (CGP55845) –
Blasco-Serra et al. (2020) [89]	Sleep (SWS-I *, SWS-II *, REM *) Atonia *	#	#	-
Acidic Saline				
Liu et al. (2014) [97]	MPWT * EPM * OFT * SCT * SPT * FST *	*	-	-
Liu et al. (2017) [93]	MPWT * EPM * SPT * FST *	*	-	Pregabalin * Duloxetine * Diazepam *
Murasawa et al. (2020) [95]	MPWT * OFT * EPM *	*	-	Mirogabalin *
Lottering & Lin (2021) [98]	MPWT * TWL * OFT * FST *	*	-	TRPV1 Knockout * Electroacupuncture *
Wang et al. (2021) [96]	MPWT * EPM * Burying *	*	-	Mossy cell activation *
Álvarez-Pérez et al. (2022) [65]	MPWT * TWL * OFT ✓ Dark/light box ✓ FST *	*	-	Pregabalin *
Pratt et al. (2013) [99]	PEAP ✓ Learned avoidance ✓ Avoidance of voluntary activity ✓	✓	✓	-
Heimfarth et al. (2020) [94]	MPWT * Grip strength ✓ OFT ✓ NOR # EPM *	*	#	Myrtenol ✓ Myrtenol + β-ciclodextrin (βCD) * Pregabalin #
Murasawa et al. (2021) [100]	MPWT * Y-maze * NOR * MWM * Step-through passive avoidance*	-	*	Mirogabalin *
Sutton & Opp (2014a) [101]	MPWT * Sleep (transitions*, W ✓, NREM ✓, REM *)	#	#	-
Sutton & Opp (2014b) [103]	MPWT * Sleep fragmentation *	#	#	-
Wei et al. (2019) [102]	Sleep (W *, NREM1 *, NREM2 *, transition sleep *, REM ✓)	#	#	-
Fatigue-Enhanced Muscle Pain				
Pratt et al. (2013) [99]	PEAP ✓ Learned avoidance ✓ Avoidance of voluntary activity ✓	✓	✓	-
Subchronic Swim Stress				
Dhir & Kulkarni (2008) [120]	Tail-flick latency * RotaRod * FST (model induction) * EPM ✓ Locomotor activity # Mirror chamber *	#	-	Venlafaxine *
Sachdeva et al. (2010) [115]	Tail-flick latency * FST (model induction) * Fatigue (grooming initiation) * Mirror chamber * EPM *	*	-	Epigallocatechin gallate (EGCG) *
Trivedi & Sharma (2011) [117]	Tail-flick latency * FST (model induction) * Locomotion * RotaRod * Mirror chamber * EPM ✓	*	-	Glycyrrhiza glabra* Fluoxetine*
Saha (2011) [116]	Escape behavior * OFT ✓ EPM ✓	*	-	-
Li et al. (2017) [113]	MPWT * Inclined plane ✓ FST * SPT * EPM ✓	*	-	Imipramine * Ifenprodil *
Nazeri et al. (2018) [114]	Orofacial formalin ✓ test EPM * RotaRod ✓ Wire grip ✓	*	-	-
Chen et al. (2018) [112]	SPT * FST * TST * Inclined plane ✓	*	-	-
Zhang et al. (2020) [127]	MPWT * FST (model induction) * TWL * TST *	*	-	-
Xue et al. (2020) [121]	MPWT * TWL * EPM * OFT * SPT ✓ FST (model induction) *	#	#	-
Bagues et al. (2022) [111]	Orofacial formalin ✓ Paw formalin * Hypertonic saline stimulation * EPM ✓ Locomotor activity *	*	-	Morphine *
Nazeri et al. (2014; 2016) [118,119]	TWL * Tail-flick latency * Passive avoidance * OFT ✓	✓	*	L-Arginine * L-NAME *
Cold Stress				
Nishiyori et al. (2011) [33]	MPWT * TWL * TST ✓ EPM ✓ Locomotor activity ✓	✓	-	Milnacipran - Amitriptyline - Mianserin - Paroxetine -
Montserrat-de la Paz et al. (2015) [138]	MPWT * TWL * Tail-flick latency * Hole-board test * Traction test * Evasion test *	*	-	Gabapentin *
Lee et al. (2018) [137]	MPWT * TWL * Tail-flick latency * TST *	#	-	Valeriana fauriei *
Nasu et al. (2019) [139]	FST *	*	-	Neurotropin * Imipramine *
Sound Stress				
Green et al. (2011) [143]	Visceral hyperalgesia * Spinal hyperalgesia * EPM *	*	-	-
Golzio dos Santos et al. (2020) [145]	EPM ✓ OFT #	#	-	Riparin III
Hung et al. (2020) [144]	MPWT * MWT * TWL * OFT * EPM * Grip force *	*	-	Pregabalin – Morphine - Diclofenac -
Viero et al. (2022) [146]	MPWT * Periorbital thresholds * Grimace * OFT *	*	-	CGRP receptor antagonist (olcegepant; BIBN4096BS *)

**Table A2:** Primary model induction protocols for preclinical fibromyalgia.

Model	Methods
Reserpine/Biogenic Amine Depletion [29]	Subcutaneous reserpine (1 mg/kg) daily, for 3 consecutive days
Acidic saline [30]	Two intramuscular injections of 4.0 pH saline into the gastrocnemius, 5 days apart
Fatigue-enhanced muscle pain [31,32]	Fatigue protocol (wheel running or direct muscle stimulation) followed by muscular insult (0.03% carrageenan, or 2 injections of 5.0 pH saline, 5 days apart)
Subchronic Swim Stress [36]	Forced swimming in cylindrical tube for 3 consecutive days (10 min on day 1, 20 min on days 2 and 3)
Cold Stress [33]	Overnight exposure to cold (−3 °C) for 15 h, then alterations in temperature every 30 min between room temperature (22 °C) and cold temperature (−3 °C) for 5 days
Sound Stress [34,35]	5 or 10 s 105 dB tone of mixed frequencies (11–19 kHz), every minute at random times, for a period of 30 min on days 1, 3, and 4.
